# Female Psychopathy and Mortality

**DOI:** 10.3389/fpsyt.2022.831410

**Published:** 2022-03-10

**Authors:** Olli Vaurio, Markku Lähteenvuo, Hannu Kautiainen, Eila Repo-Tiihonen, Jari Tiihonen

**Affiliations:** ^1^Department of Forensic Psychiatry, University of Eastern Finland, Niuvanniemi Hospital, Kuopio, Finland; ^2^Unit of Primary Care, Helsinki University Central Hospital, University of Helsinki, Stockholm City Council, Helsinki, Finland; ^3^Department of General Practice, University of Helsinki, Helsinki, Finland; ^4^Department of Clinical Neuroscience, Center for Psychiatry Research, Karolinska Institutet, Stockholm City Council, Stockholm, Sweden; ^5^Neuroscience Center, University of Helsinki, Helsinki, Finland

**Keywords:** forensic science, psychopathy, mortality, causes of death, PCL-R

## Abstract

The mortality of female psychopaths has scarcely been investigated. To estimate the association between psychopathy and mortality, data from subjects having been in forensic psychiatric assessments at Niuvanniemi Hospital during 1984–1993 were linked to the data from the National Death Registry. Sixteen psychopathic females scoring 25 points or higher in the PCL-R scale (psychopaths) were followed up for a median (IQR) 21 (17–25) years and 41 offenders scoring <25 on the PCL-R (non-psychopathic offenders) for 22 (17–25) years. In both psychopath and non-psychopath offender groups, the mortality was significantly higher (*p* < 0.01) than in the general population, being over 12-fold among psychopathic and over 6-fold among the non-psychopathic offenders.

## Introduction

Antisocial personality disorder (ASPD) predominantly affects males. The prevalence of ASPD in females is estimated to be ~2% in the general population, whereas up to 6% men meet the diagnostic criteria (1). Females with ASPD are more likely to commit non-violent antisocial acts (e.g., miss work/school, run away from home), while males with ASPD tend to be more likely involved in illegal and violent actions (2). Psychopathy is considered to be the extreme manifestation of antisocial personality disorder (3, 4). Psychopathic individuals are characterized by antisocial behavior, lack of empathy, irresponsibility, superficial charm and the tendency to be manipulative in interpersonal relations. As with ASPD, males with high psychopathy ratings are overrepresented when compared to females. Salekin et al. (5) found that in prison settings, the overall prevalence rate was about half for females (15.5%) than what is generally reported for male prisoners (25–30%). The PCL-R (6) was originally targeted at detecting psychopathy in males and not in female populations. Some studies suggest that there are in fact “male items” (Callous/Lack of empathy and Juvenile delinquency) and “female items” such as promiscuous sexual behavior (7). Also, the term “macho man” is used to describe one of the prototype characteristics to score one of the items. A recent study has shown that especially some items in the PCL-R are gender biased (8) and generally females tend to score lower on the PCL-R than males (9–11). Thus, different cut-off criteria for psychopathy have been used for males and females, with cut-offs as low as 23 being used for females while cut-offs of 25–30 are often employed in males (11–13). One of the noted differences between males and females is that while male psychopaths more frequently show antisocial and physical aggression, females tend to display a more relational and verbal form of aggression, as well as more manipulativeness and self-destructive behavior (11, 14).

The association between ASPD and premature death has been well-established in many previous studies (15, 16). The elevated mortality is suggested to be caused by a perilous style of life which is characteristic for antisocial personality. Injuries caused by accidents, fights and excessive use of substances are not uncommon and contribute to the shorter life expectancy among offenders (17). There are indications that the degree of psychopathy affects life expectancy in males (18), but such correlation is yet to be established in females. As the characteristics of psychopathy and aggressive behavior between male and female psychopaths have been shown to be somewhat different, and these factors have been associated with increased mortality, they may translate into differences in risk of mortality between male and female psychopaths (19).

## Materials and Methods

The study cohort was gathered from Niuvanniemi Hospital files of forensic psychiatric assessments (FPA) performed during the years 1984–1993 in Finland. In the course of that time, a total of 543 individuals underwent forensic psychiatric evaluation. In the Finnish judicial system, FPA is a standard procedure to evaluate the accountability of a person in court in the case of severe crimes (20).

The process, generally lasting 2 months, consists of frequent psychiatric interviews, a wide variety of psychological, somatic and other methods to evaluate the offender's mental state during the criminal offense and at the same time diagnose possible psychiatric conditions. For the examination, a wealth of information is gathered from multiple sources such as comprehensive medical and criminal records, former employers, family members and educational institutions. In repeated interviews the subject's narration is compared to these documents in order to uncover any discrepancies. As the end product of the examination, a statement of the legal accountability of the individual is given to the court. According to Finnish criminal law, the offender is not criminally responsible if, at the time of the crime, the individual was not able to understand the factual nature or unlawfulness of his/her act, or his/her ability to control his/her behavior was decisively weakened due to mental illness, severe mental deficiency, a serious mental disturbance, or a serious disturbance of cognition. Individuals who are legally accountable are sentenced to prison, whereas those who are not guilty by reason of insanity or intellectual disability, are committed to involuntary treatment (20).

In the whole sample of 543 individuals who underwent FPA, 454 (83.6%) of the offenders were male and only 89 (16.4%) were female. For the females, the mean age at the time of FPA was 35.7 years (SD 12.5 years). The ages of the offenders ranged from 16 to 70 years. In 30 (33.7%) cases, the primary index crime was a violent act leading to death (murder, manslaughter) and in 31 (34.8%) the offenders were charged with a non-lethal offense (assault, aggravated assault). Of the remaining, 20 had (22.5%) committed arson, 6 (6.7%) crime against property, and in two cases (2.2%) some other kind of offense (e.g., drug related crimes). One person had a homicide as a second charge, and 19 (21.3%) had perpetrated a non-lethal violent crime as a second offense as well. At the time of data gathering, all of the individuals were screened for ASPD according to DSM-IV criteria, whether or not they had been diagnosed with ASPD during the original FPA. The screening was done by a single trained and experience forensic psychiatrist (O.V.) using the information from the FPA. Of all the females who underwent FPA, 45 (50.6%) were diagnosed with a personality disorder and almost half of them (21) met the diagnostic criteria for ASPD. Psychotic disorders (schizophrenia, schizoaffective disorder or delusional disorder) were diagnosed in 20 (22.5%) cases. The remaining were either intellectually disabled (5.6%), suffered from an organic brain syndrome (5.6%), were diagnosed with a mood disorder (2.2%) or did not receive any psychiatric diagnosis (10.1%). It is notable that sixty percent of the offenders had definite alcohol addiction although in one third of all the cases, the presence of dependence was controversial due to missing information. In this sample, the use of illicit substances was only sporadic.

The exclusion criteria for the study were major mental illness (schizophrenia, schizoaffective disorder or delusional disorder), low intelligence quotient (WAIS <70), organic brain syndrome (dementia or brain damage) or age under 18 years. The rationale for excluding chronically psychotic, organically affected, and mentally handicapped individuals was to eliminate distracting factors such as aggression caused by paranoid delusions or poor judgment and the inherently increased mortality seen in schizophrenia and intellectual disability (21, 22). In the same manner, for example damage to the frontal lobe could alter a person's behavior to resemble the impulsivity and blunted emotions seen in psychopaths. From the initial group of 89 female offenders, 32 subjects were excluded. A total of 20 individuals were eliminated for major mental illness, five for intellectual disability, five for organic brain disorder and two for being under 18 years of age. Individuals under 18 years of age were excluded, since diagnosing personality disorders in individuals younger than 18 years is not recommended because their personality may still mature. The remaining 57 females were categorized into low and high psychopathy groups by using the Psychopathy Checklist-Revised (PCL-R).

The scoring of the PCL-R is based on semi-structured interview and official documents, such as criminal records which are then compared to a person's subjective narration. There are 20 items and each one is scored on a scale from zero to two. The value 0 indicates “does not apply at all”, 1 and 2 points refer to “partial match” and “good match”, respectively. The items are superficial charm, grandiosity, need for stimulation, pathological lying, manipulativeness, lack of remorse, shallow affect, lack of empathy, parasitic lifestyle, poor behavioral controls, promiscuity, early behavior problems, lack of long-term goals, impulsivity, irresponsibility, failure to accept responsibility, many short-term marital relationships, juvenile delinquency, revocation of conditional release and criminal versatility.

The argument for using PCL-R was that it is the most widespread and reliability proven method to evaluate psychopathy and can be applied to cases where only records or other written material are available (23). Initially PCL-R was validated only in male populations, but further studies have demonstrated it to be executable in female populations as well (7, 11). The PCL-R scores were assessed by a single person (O.V.) with proven interrater reliability (Intraclass correlation coefficient, ICC 0.89).

The cohort was divided into two groups based on PCL-R score. A cut-off score of 25 points was used, a conventional procedure in many European countries, instead of the US cut off score of 30, although lower cut off scores have also been suggested for females (9–12). The PCL-R scores ranged from 1 to 32.2 points, the average for the total female cohort was 16.8 points. Sixteen subjects (28.1%) scored 25 points or higher, the mean age for this group was 30.6 years. For those scoring below 25 points the mean age was slightly higher, 34.4 years (*n* = 41). The subjects with high PCL-R scores—and in fact all of the subjects—were Caucasian, native Finnish citizens. Data on mortality during years 1984–2013 was obtained from Statistics Finland.

### Statistical Analysis

The descriptive statistics were presented as means with standard deviation (SD), as medians with interquartile range (IQR) or as counts with percentages. Kaplan-Meier's method was performed to estimate cumulative survival. The ratio of observed to expected number of deaths, the standardized mortality ratio (SMR) for all-cause deaths, was calculated for both study groups using the subject-years methods with 95% confidence intervals (CI), by utilizing the national database of Statistics Finland, assuming a Poisson distribution. The expected number of deaths was calculated on the basis of sex-, age- and calendar-period-specific mortality rates in the Finnish population.

## Results

**Figure 1:**
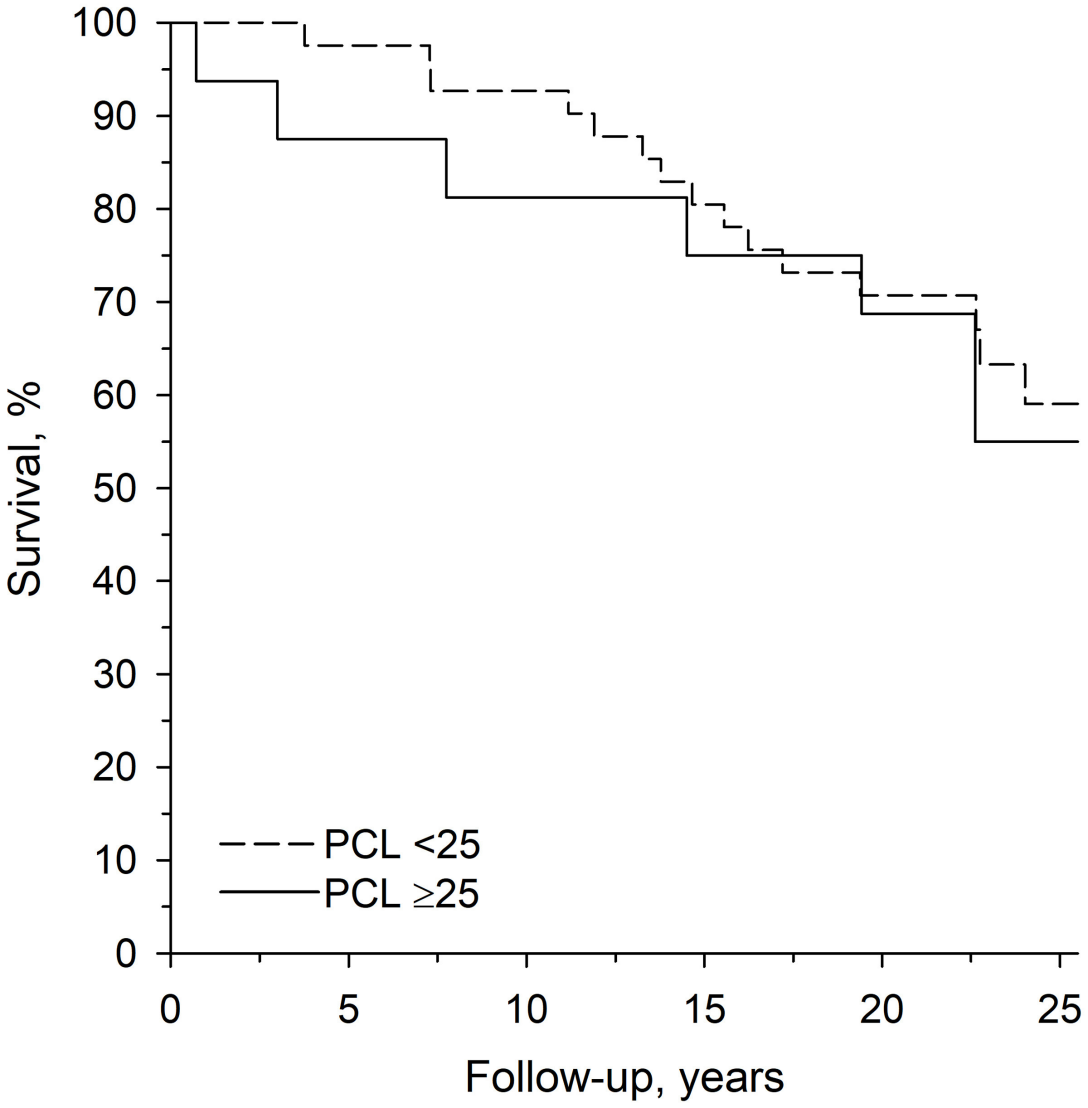
Age-adjusted survival curves for low and high psychopathy groups.

A total of 22 deaths occurred during the follow-up period. The mean age of the subjects at the time of death was 52.7 years (SD 14.9, range 22–83). On median (IQR), the subjects were followed for 22 years (17–25); the low psychopathy group for 22 years (17–25) and the high psychopathy group for 21 years (17–23). The SMR for the low psychopathy group was 6.30 (3.86–10.29) and 12.87 (5.78–28.65) for the high psychopathy group (*p* = 0.13 for difference between these groups, Figure 1). The mortality was significantly higher in both study groups when compared to the general population (*p* < 0.001).

For all the fatalities natural causes accounted for 63.6% (*n* = 14) and unnatural causes 36.4% (*n* = 8) of the cases (Table 1). For the low psychopathy group, a total of 16 deaths were recorded and the causes of death for this group were cerebrovascular disease (18.8%, *n* = 3), cardiovascular disease (12.5%, *n* = 2), cancer (12.5%, *n* = 2), liver disease (12.5%, *n* = 2), accidents (12.5%, *n* = 2), intoxication (12.5%, *n* = 2), pulmonal disease (6.3%, *n* = 1), suicide (6.3%, *n* = 1), homicide (6.3%, *n* = 1). For the high psychopathy group, a total of six deaths were recorded and the causes of death were cancer (33.3%, *n* = 2) and cerebrovascular disease, liver disease, suicide and drowning (16.7%, *n* = 1 for each).

**Table 1:** Causes of death in low and high psychopathy groups.

	**PCL <25 (low)**	**PCL 25+ (high)**	**Total**
Cancer	2	2	4
Pulmonal disease	1	0	1
Cardiovascular disease	2	0	2
Cerebrovascular disease	3	1	4
Liver disease	2	1	3
Suicide	1	1	2
Homicide	1	0	1
Drowning	0	1	1
Accident	2	0	2
Intoxication	2	0	2
Total	16	6	22

## Discussion

It has been established in many previous studies that antisocial personality lifestyle predisposes to premature death in both male and female populations (15–18, (24, 25)). Our study consolidates this finding but also adds new supplemental information on the mortality rates of antisocial and particularly psychopathic females. In fact, to our knowledge, this is the first study focusing on mortality among female psychopaths. However, a previous study from the Netherlands has explored the effectiveness of risk assessment tools in predicting recidivism and mortality in a female forensic psychiatric cohort, and in that study higher scores for the PCL-R Facet 1 (Interpersonal) were associated with reduced mortality. In their study, the mean age of death for the deceased was 44.6 years, markedly lower than reported in our study (52.7 years), and at least 21% had died due to suicide (not all causes of death were known) as opposed to the 9% in our study. As they did not exclude females with major psychiatric disorders, such as schizophrenia, which are themselves associated with markedly increased mortality and rate of suicides (22), the lower mean age of death and high prevalence of suicides reported may be a product of skewing caused by these major psychiatric disorders.

Although in our study we were not able to study the effects of the PCL-R facets, we found that higher scores on the PCL-R in general were associated with higher mortality. Thus, one of the key findings was that the mortality for the low PCL-R offender group was over six times higher than the general population, and even higher, 12-fold for the high PCL-R group. Our previous findings with a similarly constructed and analyzed cohort of males demonstrated three times higher mortality among non-psychopathic males with ASPD and almost 5-fold higher mortality among males with psychopathy when compared to the general population (18). In the psychopath group, unnatural causes of death were more frequent (65%) than in the non-psychopath group (43%). The causes in the psychopath group were also more violent: suicides, homicides and accidents were the leading causes, along with intoxications (18). In females the percentage of unnatural deaths was lower than in males, but similar between both groups of different levels of psychopathy (37.5% for low psychopathy and 33.3% for high psychopathy).

Although a large proportion of the fatalities were attributed to natural causes, a considerable amount of the deaths could be traced to an antisocial lifestyle. The impulsivity and lack of responsibility extend to health also; for example, cigarette smoking is very commonplace among psychopathic females (26), leading to a high rate of lung diseases, and the heavy use of alcohol account for liver diseases and different forms of malignant neoplasms. An unstable lifestyle delays contacts with healthcare providers and makes medical appointments and follow-up visits unfeasible. Antisocial behavior frequently derives from deficient childhood circumstances, and this poses a threat to somatic wellbeing later in life (27). Individuals with high degree of psychopathy may bear an increased genetic burden for addictions and violence (28) and childhood adverse events make matters even worse. High mortality may be also partly a result of the greater vulnerability of females to the physical damages of alcohol and other psychoactive substances (29). When compared to males, females usually have better social networks, which can be seen as a protective factor. Due to the exploitative nature of the disorder, psychopathic females tend to have less trusting and fewer long-lasting human relationships and, therefore, less support. Psychopathy in females is a relatively rare phenomenon when compared to males, which induced difficulties with finding an adequate number of females with high PCL scores. Despite the small number of subjects, we found the typical causes of death as expected in an antisocial cohort. However, in our study group antisocial lifestyle did not cause as many unnatural deaths as one might expect, and there is a clear difference between males and females in this respect (16). It might be a feature typical of females and needs further investigation. Although effective treatment options for adult antisocial psychopaths are scarce (30), many of those suffering from its consequences could benefit from information about common health issues and targeted measures to alleviate them. There is also some evidence that early interventions, such as parent management training (PMT) or adequate treatment of conduct disorder, may be beneficial in preventing the possible later life complications in children and young people (CYP) at risk of psychopathy (30).

The causes of death in the Finnish general population mirror those of Western European countries, and according to a WHO report in 2017, life expectancy at birth was 1 year above the EU average (https://www.euro.who.int/__data/assets/pdf_file/0011/355979/Health-Profile-Finland-Eng.pdf). Thus, the mortality in Finland in general is comparable to that of the rest of Europe. However, as the mortality of female psychopaths has not been widely studied in many other countries, it is difficult to evaluate whether there are differences between countries in this regard. As many of the deaths observed in the female psychopathy group were related to alcohol use-related causes of death, general trends in alcohol use between countries may also affect the mortality of female psychopaths. Thus, in countries where female alcohol use is low, the mortality rate and causes of deaths may be different also for female psychopaths.

The main limitation of this study is the small sample size, which resulted from the small number of female psychopaths in the initial cohort. Even though a PCL-R cut off point of 25 points was applied, only 16 female subjects (1.6 per year) were caught during the years 1984–93. For the males during the same period, the number of individuals caught was 100 (10 per year). Also of note is that in this study the PCL-R rating was done *post-hoc* by using only written information from the FPA. In this study, only the ultimate endpoint of death was observed, without information on the circumstances leading to either the death or survival of the subjects. For those reasons, caution should be taken when interpreting the results. Also, as the subjects were collected from forensic psychiatric assessments, only individuals who committed crimes serious enough to warrant FPA could be included in this study. Thus, individuals not being sent for FPA, for example due to not getting caught, could not be included. The strengths of this study are the considerable follow-up time, the fact that the study participants were all rated by the same individual and that a plethora of information was available for the PCL-R rating.

In general, female psychopathy is understudied and merits further research. It would be interesting to study whether there are different factors behind the increased mortality observed in female and male psychopaths. However, larger samples are needed to dissect more distinct factors. Artificial intelligence-based methods may provide a way to disentangle these differences more efficiently than traditional statistical methods, and could provide an interesting modality for future studies.

## Data Availability Statement

The raw data can be requested from the pertinent registry holders. Further enquiries can be directed to the corresponding author, Dr. Olli Vaurio, olli.vaurio@niuva.fi.

## Ethics Statement

Ethical review and approval was not required for the study on human participants in accordance with the local legislation and institutional requirements. Written informed consent from the participants' legal guardian/next of kin was not required to participate in this study in accordance with the national legislation and the institutional requirements.

## Author Contributions

OV and JT: study design. OV and HK: data analysis and data interpretation. OV and ML: manuscript preparation and editing. JT: supervision. All authors contributed to the article and approved the submitted version.

## Funding

This study was funded by the Finnish Ministry of Social Affairs and Health through the developmental fund for Niuvanniemi Hospital, Kuopio, Finland. The funder of the study had no role in study design, data collection, data analysis, data interpretation, or writing of the report.

## Conflict of Interest

The authors declare that the research was conducted in the absence of any commercial or financial relationships that could be construed as a potential conflict of interest.

## Publisher's Note

All claims expressed in this article are solely those of the authors and do not necessarily represent those of their affiliated organizations, or those of the publisher, the editors and the reviewers. Any product that may be evaluated in this article, or claim that may be made by its manufacturer, is not guaranteed or endorsed by the publisher.
